# Daily transcriptome changes reveal the role of nitrogen in controlling microcystin synthesis and nutrient transport in the toxic cyanobacterium, *Microcystis aeruginosa*

**DOI:** 10.1186/s12864-015-2275-9

**Published:** 2015-12-16

**Authors:** Matthew J. Harke, Christopher J. Gobler

**Affiliations:** School of Marine and Atmospheric Sciences, Stony Brook University, 239 Montauk Hwy, Southampton, NY 11968 USA

**Keywords:** *Microcystis*, Transcriptome, Microcystin, Nitrogen

## Abstract

**Background:**

While transcriptomics have become a valuable tool for linking physiology and ecology in aquatic microbes, the temporal dynamics of global transcriptomic patterns in *Microcystis* have rarely been assessed. Furthermore, while many microbial studies have explored expression of nutrient transporter genes, few studies have concurrently measured nutrient assimilation rates. Here, we considered how the global transcriptomic patterns and physiology of the cyanobacterium, *Microcystis aeruginosa*, changed daily as cells were grown from replete to deficient nitrogen (N) conditions and then back to replete conditions.

**Results:**

During N deprivation, *Microcystis* downregulated genes involved in photosynthesis and respiration, carbon acquisition, lipid metabolism, and amino acid biosynthesis while upregulating genes involved in N acquisition and transport. With increasing N stress, both the strength of expression and number of genes being differentially expressed increased, until N was restored at which point these patterns reversed. Uptake of ^15^N-labeled nitrate, ammonium and urea reflected differential expression of genes encoding transporters for these nutrients, with *Microcystis* appearing to preferentially increase transcription of ammonium and urea transporters and uptake of these compounds during N deprivation. Nitrate uptake and nitrate transporter expression were correlated for one set of transporters but not another, indicating these were high and low affinity nitrate transporters, respectively. Concentrations of microcystin per cell decreased during N deprivation and increased upon N restoration. However, the transcript abundance of genes involved in the synthesis of this compound was complex, as microcystin synthetase genes involved in peptide synthesis were downregulated under N deprivation while genes involved in tailoring and transport were upregulated, suggesting modification of the microcystin molecule under N stress as well as potential alternative functions for these genes and/or this toxin.

**Conclusions:**

Collectively, this study highlights the complex choreography of gene expression, cell physiology, and toxin synthesis that dynamic N levels can elicit in this ecologically important cyanobacterium. Differing expression patterns of genes within the microcystin synthetase operon in response to changing N levels revealed the potential limitations drawing conclusions based on only one gene in this operon.

**Electronic supplementary material:**

The online version of this article (doi:10.1186/s12864-015-2275-9) contains supplementary material, which is available to authorized users.

## Background

The cyanobacterium *Microcystis* commonly forms blooms in temperate, freshwater ecosystems and can produce the hepatotoxin, microcystin. Toxic blooms of this species have led to beach closures, public water emergencies, and health concerns [1–3]. Anthropogenic nitrogen (N) loading can be an important factor in the occurrence of cyanobacteria blooms caused by non-diazotrophs such as *Microcystis* spp. [4–6]. For instance, field studies have demonstrated that N loading can promote blooms of *Microcystis* [7, 8] and toxic strains of *Microcystis* can outgrow non-toxic strains under high N levels [6, 9, 10]. There appears to be a link between N and microcystin production, whereby N-deprived *Microcystis* cells display reduced transcription of microcystin synthetase genes and decreased microcystin content as compared to replete N cells [11]. This observation has been supported by culture studies [10, 12–15] and field studies that have found that increases in exogenous N concentrations have been associated with increases in microcystin in lakes [5, 11, 13–16].

While the role of phosphorus in controlling the growth and physiology of freshwater cyanobacteria has been well studied [17, 18], comparatively less is known regarding responses to dynamic levels of N. During nitrogen stress, some cyanobacteria are known to initially induce systems for uptake of multiple N-containing compounds [11, 19] and N acquired through these transporters is then converted to ammonium and assimilated through the glutamine sythetase-glutamate synthetase cycle (GS-GOGAT). When exogenous N availability becomes too low to supply internal N demands, cyanobacteria may begin to rely on internal stores of N to prolong growth and may downregulate photosynthesis [20–22]. Still, freshwater cyanobacterial studies exploring physiological linkages between gene responses to N limitation and changes in cell physiology have been limited, especially for toxic, freshwater genera such as *Microcystis*.

Transcriptomics have become a valuable tool for investigating linkages between physiology and ecology in aquatic microbes. To date, most global transcriptomic studies have examined single time points or time series studies over a day/night cycle [11, 23–29] with the exception of limited microarray studies [22, 30]. In this study, we sought to document the daily global transcriptomic patterns of *Microcystis aeruginosa* over a one week period as cells were grown from replete to deficient N conditions and returned to replete conditions. To assess the choreography of gene expression and cell physiology we examined expression patterns of microcystin synthetase genes and genes relating to N transport, carbon acquisition, and photosynthesis in unison with levels of cellular microcystin and nutrients, as well as the uptake rate of nitrogenous compounds and bicarbonate. To our knowledge, the relationship between the expression of transporter genes and the uptake of corresponding target compounds (as measured here via isotopic tracers) has never been documented in cyanobacteria.

## Methods

### Experimental design

Experiments were conducted to track gene expression patterns of *Microcystis aeruginosa* as populations were grown from N-replete conditions into low N conditions and returned to replete conditions. Experiments were performed with *Microcystis aeruginosa* clone LE-3 (Lake Erie, USA) [31] maintained in BG-11 medium illuminated by fluorescent lights that provided a light intensity of ~100 μmol quanta m^−2^ s^−1^ on a 14:10 light/dark cycle at 25 °C. Treatments included N replete (0.1 M nitrate) cultures (*n* = 3) to serve as a control and a set of cultures that began with 50 μM nitrate (*n* = 3). All cultures were inoculated into 2 L Erlenmeyer flasks containing 1 L media with 10^6^ cell mL^−1^ harvested from a replete culture by centrifugation (2000 × g for 10 min) [11]. Cultures were monitored daily for cell density, in vivo chlorophyll *a* fluorescence, photosynthetic efficiency, total microcystin concentrations, ^15^N and ^13^C uptake, and dissolved and total nutrient concentrations (see below for methods) at the same time daily to avoid diel changes in gene expression and cell physiology [32]. In addition, for transcriptomic sequencing, 50 mL aliquots of each replicate in each treatment were centrifuged for 10 min. at 2000 × g, immediately flash frozen in liquid N, and stored at −80 °C. Cultures were grown semi-continuously; after each daily sampling, N-replete and N-deplete cultures were diluted to 10^6^ cells mL^−1^ with BG-11 medium containing 0.1 M nitrate or no N, respectively, causing increasing N starvation in N-deplete cultures. When photosynthetic efficiency was significantly depressed in the N-deplete cultures after 5 days, a re-feed was conducted by adding in 25 μM nitrate daily after samples were collected, a level commonly achieved in dynamic lake environments during blooms [7, 33], until the experiment ended on day 8.

### Sample analysis

Lugol’s iodine preserved cells were enumerated using a Beckman Coulter Multisizer™ 3 Coulter Counter® with a 50 μm aperture which allowed cell densities to be quantified with a relative standard deviation of 3 % [11]. Nitrate was analyzed by reducing the nitrate to nitrite using spongy cadmium as per Jones [34]. Ammonium and phosphate were analyzed using techniques modified from Parsons et al*.* [35]. Total dissolved N and P were analyzed using persulfate digestion techniques from Valderrama [36]. Urea was analyzed according to Price and Harrison [37]. These nutrient analyses provided 100 ± 10 % recovery of standard reference material (SPEX CertiPrep™) for nitrate, ammonium, phosphate, total dissolved N, and total dissolved P. Whole water samples were analyzed for the hepatoxin microcystin by first freezing samples at −20 °C for 24 h and then lysing the cells using an Abraxis QuikLyse™ Cell Lysis kit for Microcystins/Nodularins ELISA Microtiter Plate according to the manufacturer’s instructions. Lysed samples were then analyzed with a colorimetric immunoassay using an Abraxis Microcystins/Nodularins (ADDA) ELISA Kit according to the manufacturer’s instructions [38]. This method provided an analytical precision of ±2 % and a 96 ± 2 % recovery of spiked samples. Maximum quantum efficiency of photosystem II (F_v_/F_m_) was estimated from in vivo (F_i_) and DCMU (3,4-dichlorophenyl-1,1-dimethylurea)-enhanced in vivo fluorescence (F_m_) of each replicate experimental sample on a Turner Designs TD-700 fluorometer (EM filter of >665 nm and EX filter of 340–500 nm) using blank corrections from BG-11 media. Our prior research has demonstrated that F_v_/F_m_ is a sensitive diagnostic of N-limitation in the strain of *Microcystis* studied here [11].

### Nitrogen and bicarbonate uptake

Tracer experiments were conducted with ^15^N and ^13^C to determine rates at which N- and C-containing compounds were being assimilated by *Microcystis* and assess the relationship between transcript abundance of specific transporters and actual uptake rates. Uptake rates of nitrate, ammonium, urea and bicarbonate were measured using additions of highly enriched (>98 %) ^15^N-nitrate, ^15^N-ammonium, ^15^N-urea, and ^13^C-sodium bicarbonate [39]. Actual concentrations of nitrate, ammonium, and urea were measured as described above whereas levels of bicarbonate were calculated using the software program, CO2SYS (http://cdiac.ornl.gov/ftp/co2sys/) based on infrared analysis of total dissolved inorganic carbon with a PP Systems EGM-4 Environmental Gas Analyzer and pH measurements with an Orion Star 3 pH meter. All isotope additions were <10 % of ambient concentrations. Incubations were performed in 50 ml acid-washed polycarbonate flasks under the same light and temperature conditions used for semi-continuous cultures for 60 min, after which water was filtered onto pre-combusted (2 h @ 450 °C) EMD Millipore APFB glass fiber filters. Subsamples of the initial water sample were also filtered onto pre-combusted EMD Millipore APFB glass fiber filters to determine the natural abundance of ^15^N and ^13^C within cells prior to enrichment. Filters were pelletized in tin discs and analyzed at the U.C. Davis Stable Isotope Facility (Davis, CA) using an Elementar vario Micro Cube elemental analyzer (Elementar Analysensysteme GmbH, Hanau, Germany) interfaced to a PDZ Europa 20–20 isotope ratio mass spectrometer (Sercon Ltd., Cheshire, UK). Uptake rates were calculated using equations from Orcutt et al. [40]. Rates were considered net uptake as they were not corrected for the effects of isotope dilution [41]; however, uptake, release, and subsequent re-uptake of compounds during incubations was likely limited given the brief incubation period (60 min).

### RNA isolation and sequencing

RNA was extracted from triplicate biological replicate samples. A RNeasy Mini Kit with RNAprotect Bacteria reagent (QIAGEN) was used to isolate RNA in a similar manner to that outlined in Ilikchyan et al. [42]. Briefly, 2 mL of RNAprotect Bacteria reagent was added to cell pellets and gently vortexed to resuspend pelleted material. The mixture was then allowed to incubate for 5 min. at room temperature and then centrifuged for 10 min. at 2000 × g. The supernatant was removed by pipet and then 200 μL of TE buffer with lysozyme (SIGMA) and proteinase K (QIAGEN) was added. The pellet was resuspended by pipetting and allowed to incubate for 10 min. at room temperature. The remainder of the RNeasy Mini Kit protocol was then followed according to the manufacturer’s instructions. To remove any traces of contaminating genomic DNA, on-column DNase digestion was performed on RNA samples using RNase-free DNase (QIAGEN) according to the manufacturer’s instructions. Ribosomal RNA was removed from total RNA (~3 μg) using an Epicenter® Ribo-Zero™ Magnetic Kit (Bacteria) according to the manufacturer’s instructions. After rRNA depletion, samples were purified using a Qiagen RNeasy MinElute Cleanup Kit according to the instructions outlined by the Epicentre Ribo-Zero™ Magnetic Kit. Post-digested RNA was assessed for quantity and quality with an Agilent Bioanalyzer™ (RNA Integrity >9.5 for all samples). Samples were stored at −80 °C until sequencing. Sequencing libraries were prepared using a TruSeq™ RNA Sample Preparation Kit v2 (Illumina®) according to the manufacturer’s instructions, skipping the poly-A pull-down step. Library prep and sequencing of 100 bp single-end reads per library was performed by the JP Sulzberger Columbia Genome Center (New York, NY) on an Illumina® HiSeq 2000 system. The Illumina® sequences reported in this paper have been deposited in the National Center for Biotechnology Information’s Sequence Read Archive (accession no. SRP056420).

### Read mapping and analysis

Prior to read mapping, raw reads were initially characterized with FastQC (http:// http://www.bioinformatics.bbsrc.ac.uk/projects/fastqc/) and were trimmed to remove ambiguous and low quality reads with Trimmomatic v0.32 [43]. Surviving reads were mapped to the reference genome *Microcystis aeruginosa* NIES-843 [44] using RSEM v1.2.19 [45] with Bowtie 2 [46] with parameters recommend by the RSEM authors. Differential expression between the treatment and reference condition was computed with EBseq [47] within RSEM. Comparisons were accepted as significant with false discovery rate (FDR) controlled at 0.05 (in EBseq PPDE ≥ 0.95). Differentially expressed genes were assigned functional categories based upon categories found in CyanoBase for the *Microcystis* NIES-843 genome (http://genome.microbedb .jp/cyanobase/Microcystis). The blastp suite (http://blast.ncbi.nlm.nih.gov) and CombFunc [48] were used to elucidate putative functions of hypothetical genes using an E-value cutoff of 1e^−5^.

### Statistical analysis

Differences in photosynthetic efficiency, cellular chlorophyll, and total microcystins between the control and low N treatment were analyzed using two-way repeated-measures ANOVAs (ANOVARs). Each experimental flask was considered a subject for these analyses and factors were nutrient condition (control or low N) and time. Post hoc comparisons were conducted with the Holm-Sidak method. Correlations between transcript abundance (log_2_ fold change) and uptake of C and N compounds were calculated using a Spearman Rank Correlation coefficient. All statistical analysis were conducted in SigmaPlot version 11.0 Build 11.1.0.102 and statistical results were considered significant at an α <0.05.

## Results

### Differential growth among treatments

**Fig. 1:**
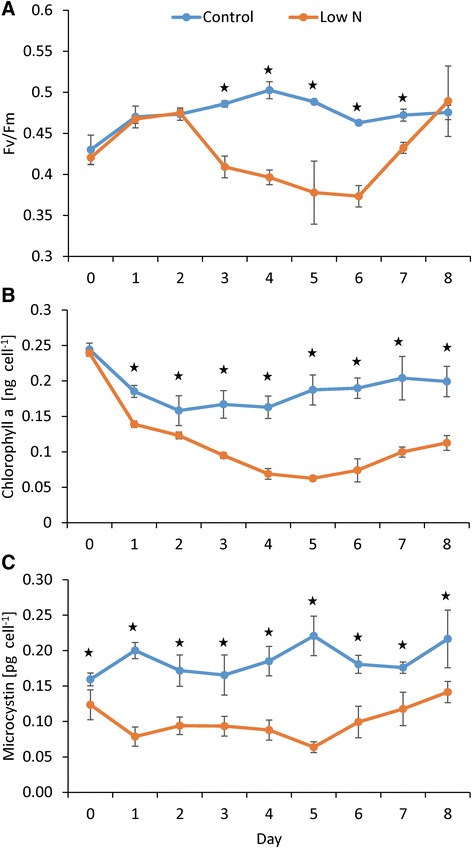
Photosynthetic efficiency, chlorophyll, and microcystin trends during the experiment. Photosynthetic efficiency (**a**), total chlorophyll *a* (**b**) and microcystin (MC-LR equivalents) concentrations per cell (**c**) during the experiment. Nitrogen refeed occurred on day 5. *Error bars* represent the standard deviation between biological replicates (*n* = 3). *Stars* indicate significant differences between treatments (*p* <0.001, two-way ANOVAR)

Multiple characteristics of *Microcystis* cultures changed as they grew in the absence of N. Although the low N culture began with 50 μM nitrate and 19 μM ammonium, concentrations were reduced to <1 μM by 24 and 48 h, respectively, and remained at this level until the re-addition of nitrate (day 5) when nitrate concentrations exceeded 7 μM but ammonium remained <1 μM (Table 1A, B). Urea concentrations were at or near sub-micromolar concentrations throughout the experiment (Table 1C). While photosynthetic efficiency (F_v_/F_m_) remained elevated in control cultures during the experiment (0.47 ± 0.2), it declined in the low N treatment and was significantly lower than the control on days 3–7 (*p* <0.001, two-way ANOVAR), reaching 0.38 ± 0.04 on day 5 after which nutrients were added (Fig. 1a). F_v_/F_m_ was unchanged on day 6 but recovered thereafter matching the control cultures by day 8 (0.49 ± 0.04; Fig. 1a). Cellular chlorophyll *a* displayed a similar patterns being unchanged in control cultures throughout the experiment (0.19 ± 0.03 ng chla cell^−1^; Fig. 1b) but significantly declining to a minimum of 0.06 ± 0.00 ng chla cell^−1^ in low N cultures (*p* <0.001, two-way ANOVAR, Fig. 1b). Upon restoration of N to the low N culture (day 5) cellular chlorophyll *a* began to recover (0.11 ± 0.01 ng chla cell^−1^; Fig. 1b). Total microcystin concentrations were similar throughout the experiment in the control whereas in the low N treatments, concentrations of microcystin declined two-fold from 0.12 ± 0.03 pg MC-LR equivalents cell^−1^ to 0.06 ± 0.01 pg MC-LR equivalents cell^−1^ on day 5 (*p* <0.001, two-way ANOVAR; Fig. 1c). When N was restored (day 5), microcystin levels recovered to concentrations similar to the control cultures and were statistically greater on days 7 and 8 relative to day 5 (*p* <0.001, two-way ANOVAR, Fig. 1c).

**Table 1:** Nutrient concentrations during the experiment

Timepoint	Replete	50 μM Nitrate
A		
0	12,410 (949)	11.81 (2.52)
1	13,097 (148)	7.90 (4.20)
2	12,734 (478)	0.54 (0.00)
3	12,917 (40)	BDL
4	13,612 (976)	BDL
5	14,607 (38)	BDL
6	13,517 (1330)	0.25 (0.00)
7	11,863 (2032)	1.76 (0.00)
8	13,791 (138)	7.49 (0.00)
B		
0	23.06 (4.18)	19.04 (3.00)
1	0.41 (0.10)	0.71 (0.18)
2	0.52 (0.02)	0.55 (0.09)
3	0.40 (0.01)	0.68 (0.32)
4	0.49 (0.10)	0.74 (0.19)
5	0.60 (0.14)	0.80 (0.09)
6	0.42 (0.03)	0.89 (0.35)
7	0.49 (0.11)	0.56 (0.35)
8	0.64 (0.57)	0.80 (0.31)
C		
0	1.15 (0.53)	0.21 (0.13)
1	N/A	0.38 (0.15)
2	N/A	0.26 (0.10)
3	N/A	0.19 (0.19)
4	N/A	0.24 (0.17)
5	N/A	0.42 (0.15)
6	N/A	0.36 (0.24)
7	N/A	0.27 (0.26)
8	0.90 (0.14)	0.17 (0.09)

Concentrations (μM) of nitrate (A), ammonium (B), and urea (C) during the experiment. Values in parenthesis are the standard deviation between biological replicates (*n* = 3). BDL denotes values below detection limit. N/A denotes not measured

### Transcriptomic sequencing

Transcriptomic sequencing yielded on average 38 million 100 bp reads per sample (Additional file 1: Table S1). Prior to generating read counts, Trimmomatic was run to remove ambiguous and low quality reads resulting in the removal of, on average, 5.25 % of reads per sample. Of the surviving reads, ~60 % aligned to the reference genome (*Microcystis aeruginosa* NIES-843) with the highest alignment rates occurring on day 2 (87 %; Additional file 1: Table S1). Between 282 and 2512 genes were significantly differentially expressed daily in low N cultures, with the highest number of genes expressed during N limitation (days 2–5, Additional file 2: Figure S1). After restoration of N (on day 5), the number of differentially expressed genes decreased by 57 % (Additional file 2: Figure S1). Of the differentially expressed genes, between 1 and 9 % displayed log_2_ fold change greater than two with the largest number of these highly expressed genes occurring at the time of strongest N limitation (9 %, day 5, lowest photosynthetic efficiency, chlorophyll *a* per cell; Additional file 2: Figure S1).

Within low N cultures, there was a >50 % increase in the number of genes that were differentially expressed in all functional categories of genes (as defined by Cyanobase; http://genome.microbedb.jp/cyanobase/) with the exception of genes with unknown function which only increased by 10 % by day 2 (Additional file 3: Figure S2). The largest fraction of genes responding on a categorical level were genes within the “fatty acid, phospholipid and sterol metabolism” functional grouping where the proportion of differentially expressed genes increased from 23 % on day 1 to 93 % of genes by day 3 of the experiment (Additional file 3: Figure S2). The smallest fraction of genes responding on a categorical level were genes within the “unknown” functional category (Additional file 3: Figure S2).

#### Nitrogen metabolism

**Fig. 2:**
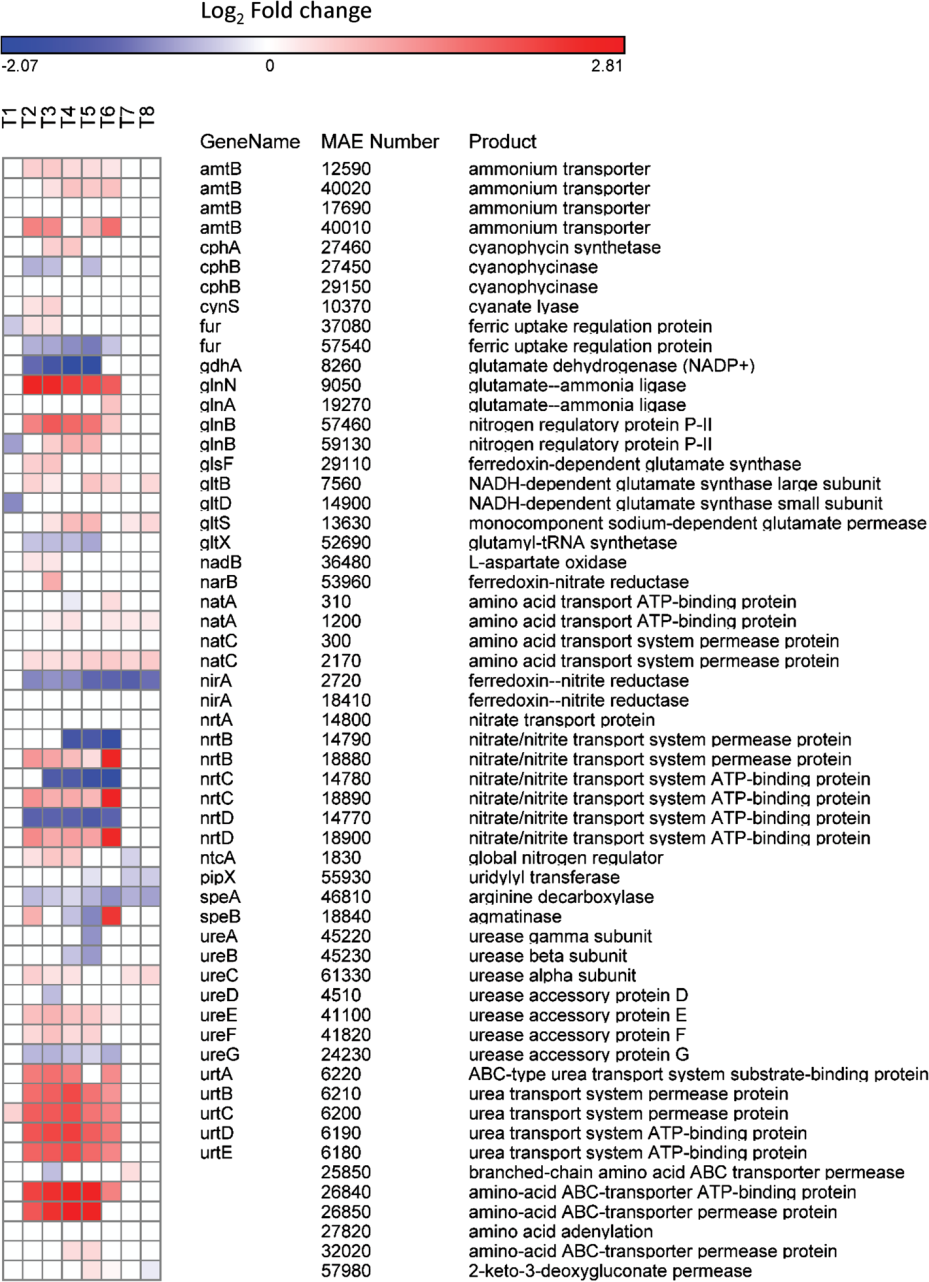
Nitrogen transport and metabolism expression patterns. Heat map of gene expression of genes involved in nitrogen metabolism and transport. *Blue colors* correspond to a decrease in transcript abundance while *red colors* correspond to an increase in transcript abundance. *White* denotes no difference from the control condition

As cells became N-limited, many genes involved in N metabolism and assimilation were differentially expressed. For instance, the global nitrogen regulatory gene *ntcA* increased in transcript abundance beginning on day 2 and peaked on day 4 (0.58 log_2_ fold change). After restoration of N, differential expression was not detected again until day 7 where it was downregulated by 0.31 fold (Fig. 2). Other regulatory genes including the nitrogen regulatory P_II_ genes (*glnB*) and the *pipX* gene were also differentially expressed during N limitation (Fig. 2). *Microcystis* has two *glnB* genes (MAE57460 and 59130) and each was upregulated after day 2 with expression disappearing after day 5 or 6 depending on the gene (Fig. 2). The gene encoding *pipX* (MAE55930) was unchanged under N limitation but decreased in transcript abundance when N was restored on days 5, 7 and 8 (Fig. 2).

Transcript abundance of genes involved in ammonium, nitrate/nitrite, amino acid and urea transport and metabolism paralleled those of nitrogen regulatory genes with increases in transcript abundance starting on day 2 and a return to similar transcript levels to the control after day 6 (one day after N restoration; Fig. 2). A more complicated response was observed for some gene sets with multiple copies. For instance, only one set of nitrate/nitrite transport systems was upregulated by N limitation (*nrtBCD*; MAE18880, 18890, 18900) while the other was downregulated (*nrtBCD*; MAE14770, 14780, 14790; Fig. 2). Furthermore, of the two nitrite reductases (*nirA*, MAE02720 and MAE18410), one was downregulated while the other displayed no change in expression relative to the control (Fig. 2). Of note, the nitrate/nitrite transporters that were upregulated under N limitation displayed even higher upregulation 24 h after nitrate was added (>2.5 log_2_ fold change, day 6) compared to previous days (<1.5 log_2_ fold change, Fig. 2). Finally, the gene involved in synthesis of the N storage product cyanophycin (*cphA*, MAE27460) was upregulated on days 3 and 4 while the gene involved in the breakdown of this product (cyanophycinase, *cphB*, MAE27450) was downregulated on days 2, 3, and 5 (Fig. 2).

#### Microcystin synthetase

**Fig. 3:**
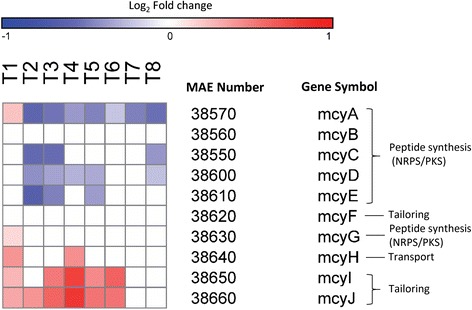
Microcystin synthetase gene expression patterns. Heat map of gene expression of genes responsible for microcystin synthetase. *Blue colors* correspond to a decrease in transcript abundance while *red colors* correspond to an increase in transcript abundance. *White* denotes no difference from the control condition

Genes involved in microcystin synthesis displayed a bifurcated response to decreasing N levels. Genes involved in tailoring and transport (*mcyHIJ*) had increased transcript abundance during N deprivation whereas genes involved in peptide synthesis (*mcyACDE*) had decreased transcript abundance (Fig. 3). After N restoration, gene expression levels (except *mcyA* which remained downregulated) became similar to control conditions in 24–48 h until day 8 when expression of *mcyCD* again decreased relative to the control. Differential expression of *mcyB* and *mcyF* was not detected. In contrast to the peptide synthetase genes (*mcyACDE*), as cells became more N limited, the microcystin tailoring and transport genes *mcyI* and *mcyJ* increased in expression from 0.32 log_2_ fold change on day 1 to 0.80 log2 fold change on day 4. When N was restored on day 5, expression decreased and became similar to control values by day 7 (Fig. 3).

#### Photosynthesis & respiration

**Fig. 4:**
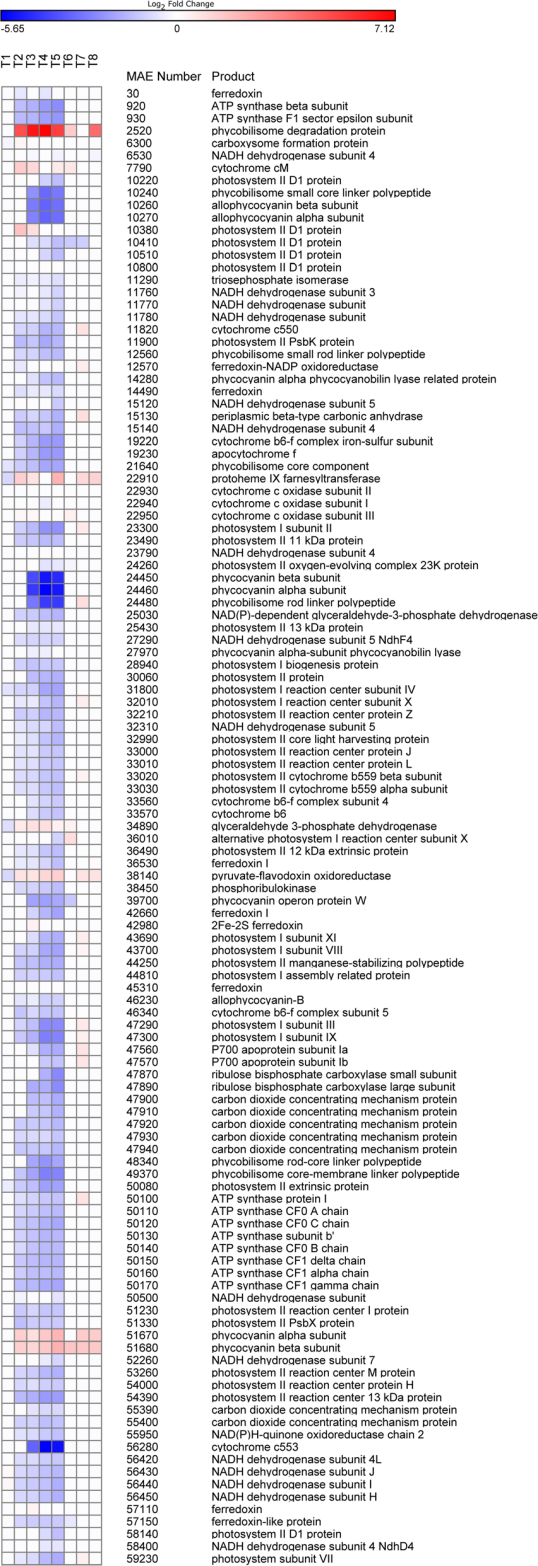
Photosynthesis and respiration gene expression patterns. Heat map of gene expression of genes involved in photosynthesis and respiration. *Blue colors* correspond to a decrease in transcript abundance while *red colors* correspond to an increase in transcript abundance. *White* denotes no difference from the control condition

When N was depleted by cultures (day 2, Table 1), >50 % of the genes involved in photosynthesis and respiration decreased in transcript abundance (Fig. 4). The number of genes and the magnitude of downregulation of these genes generally increased as cells progressed further into N limitation, peaking on day 5 (Fig. 4). After N restoration, expression of most of these genes was similar to the control condition (no differential expression) while a few began to increase in transcript abundance. Genes encoding cytochrome cM (MAE07790), protoheme IX farnesyltransferase (MAE22910), glyceraldehyde 3-phosphate dehydrogenase (MAE34890), pyruvate-flavodoxin oxidoreductase (MAE38140), two phycocyanin subunits (MAE51670 and 51680) and the phycobilisome degradation gene *nblA* (MAE02520) increased in transcript abundance starting on day 2 and then briefly returned to control values after N was restored (Fig. 4).

#### Carbon concentration and transport

**Fig. 5:**
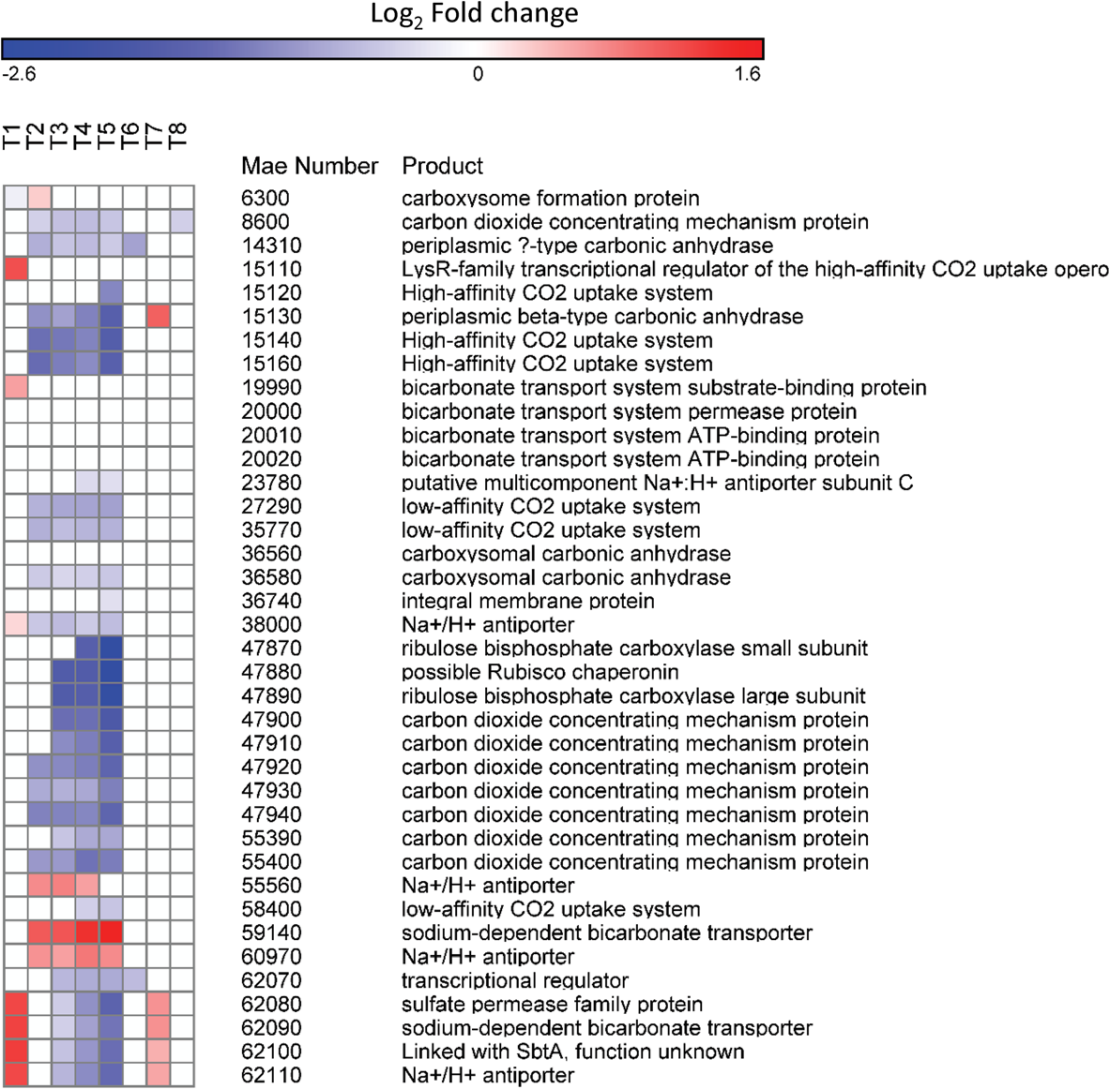
Carbon concentration and transport gene expression patterns. Heat map of gene expression of genes involved in carbon concentration and transport. *Blue colors* correspond to a decrease in transcript abundance while *red colors* correspond to an increase in transcript abundance. *White* denotes no difference from the control condition

More than 70 % of genes involved in carbon concentration and transport were downregulated beginning on day 2 with the exception of two sodium/proton antiporters (MAE55560 and 60970) and one sodium-dependent bicarbonate transporter (MAE59140) which were upregulated (Fig. 5). After N was restored, expression of many of these genes was similar to control values and on day 7, five genes were upregulated, including genes comprising a bicarbonate uptake system and a gene encoding a periplasmic carbonic anhydrase (*ecaB*, MAE15130; Fig. 5).

#### Fatty acid, phospholipid and sterol metabolism

More than 65 % of genes involved in fatty acid, phospholipid, and sterol metabolism decreased in transcript abundance as cells became N limited and when N was restored, gene expression was similar to control conditions (Additional file 4: Figure S3). Contrary to this pattern, a gene involved in lipid metabolism (MAE29450) which bears similarity to a fatty acid hydroxylase had large increases in transcript abundance during N deprivation (2.31 ± 0.24 log_2_ fold change on days 2–5; Additional file 4: Figure S3). In addition, seven other genes increased in transcript abundance during N deprivation including genes encoding membrane bound enzymes (MAE55360), fatty acid catabolism (MAE50060), glycerolipid metabolism (MAE39670 and 25770), and transferases (MAE24880 and 13400). Many of these genes returned to control conditions 24 to 48 h after N was restored (Additional file 4: Figure S3).

### ^15^N and ^13^C uptake

**Fig. 6:**
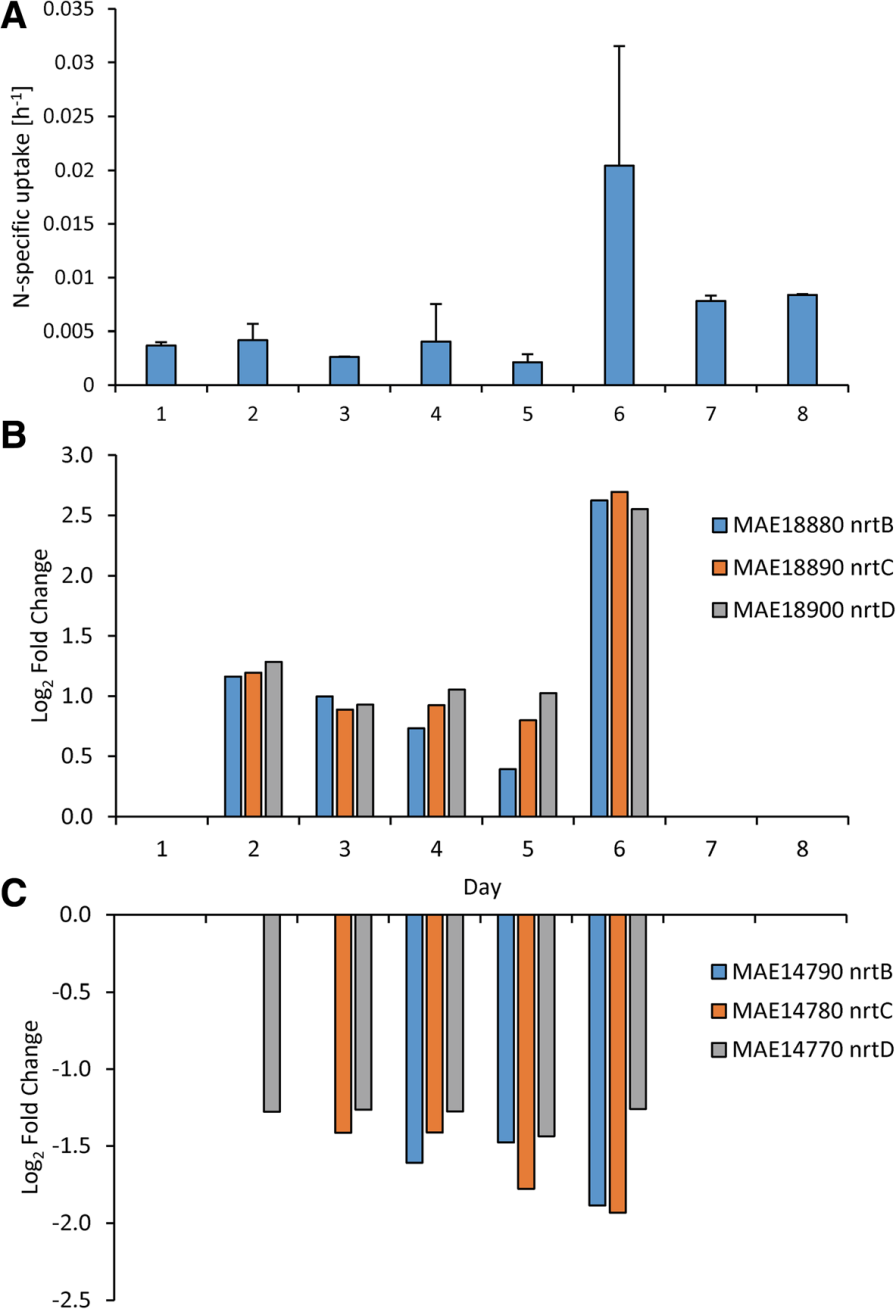
Nitrate specific uptake rates and nitrate transporter gene expression patterns. Nitrate specific uptake rates (**a**) and gene expression of nitrate/nitrite transporters (**b** & **c**) during the experiment. *Error bars* are the standard deviation between biological replicates (*n* = 3)

**Fig. 7:**
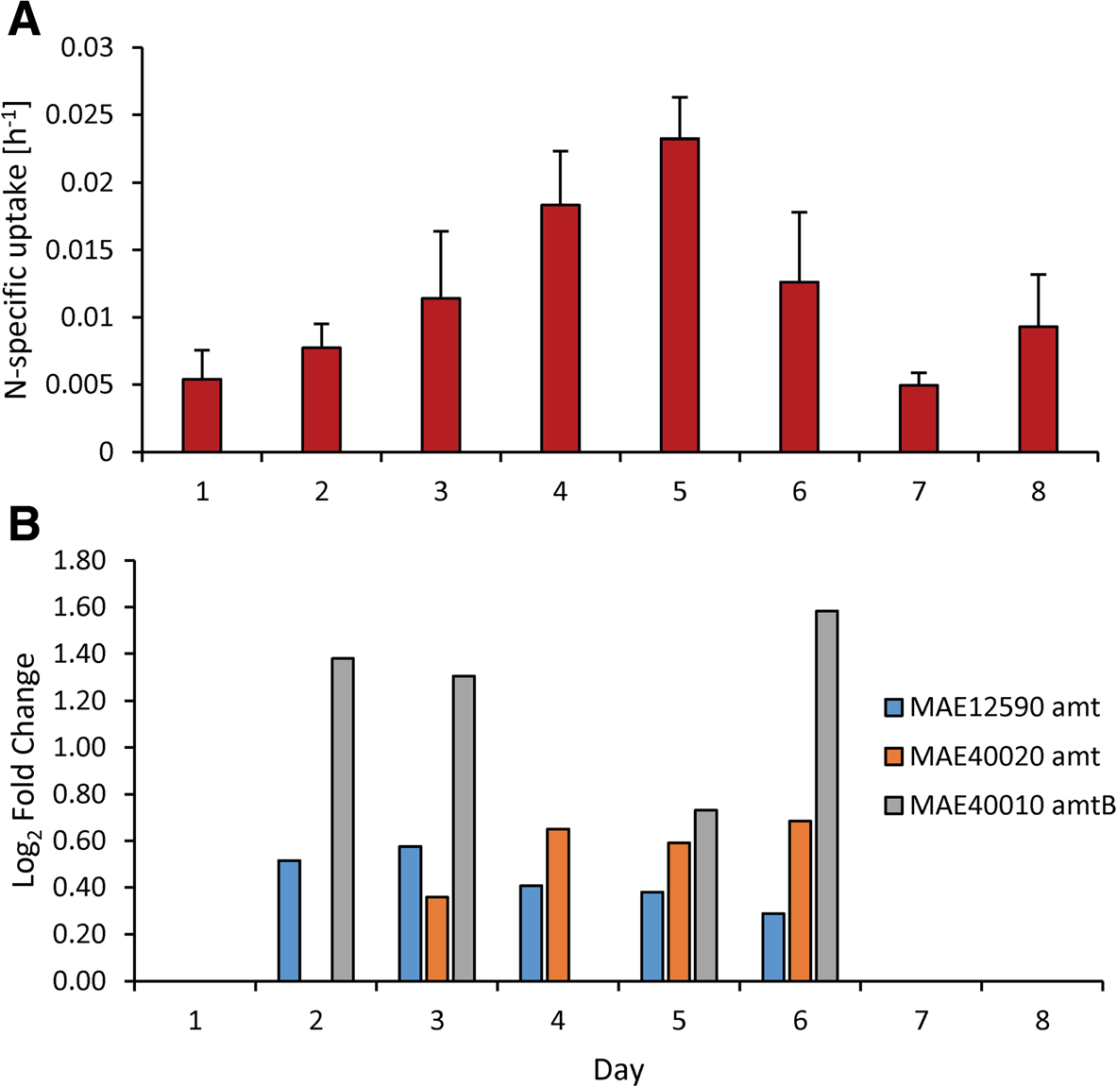
Ammonium specific uptake rates and ammonium transporter gene expression patterns. Ammonium specific uptake rates (**a**) and gene expression of ammonium transporters (**b**) during the experiment. Error bars are the standard deviation between biological replicates (*n* = 3)

**Fig. 8:**
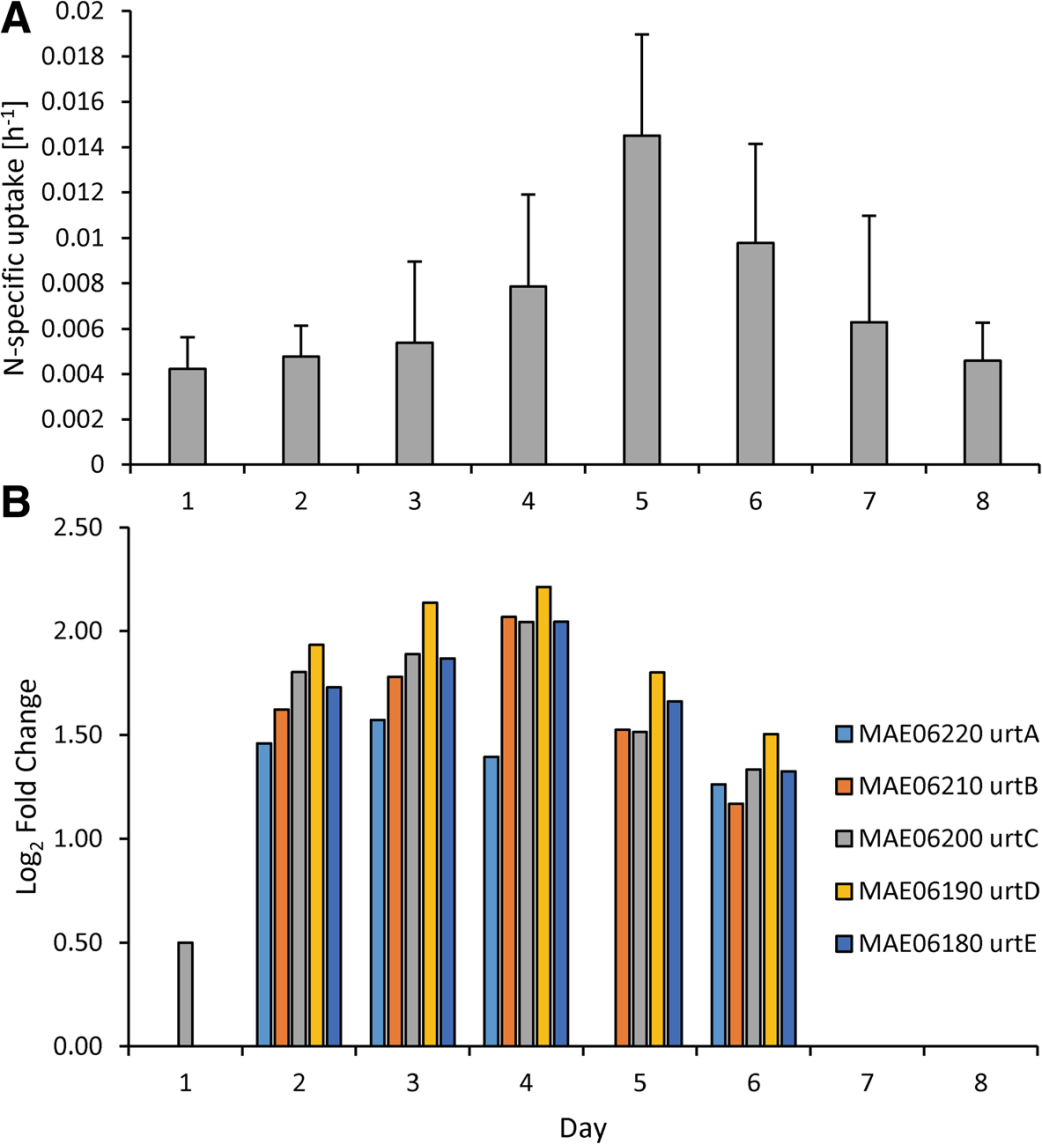
Urea specific uptake rates and urea transporter gene expression patterns. Urea specific uptake rates (**a**) and gene expression of urea transporters (**b**) during the experiment. *Error bars* are the standard deviation between biological replicates (*n* = 3)

**Fig. 9:**
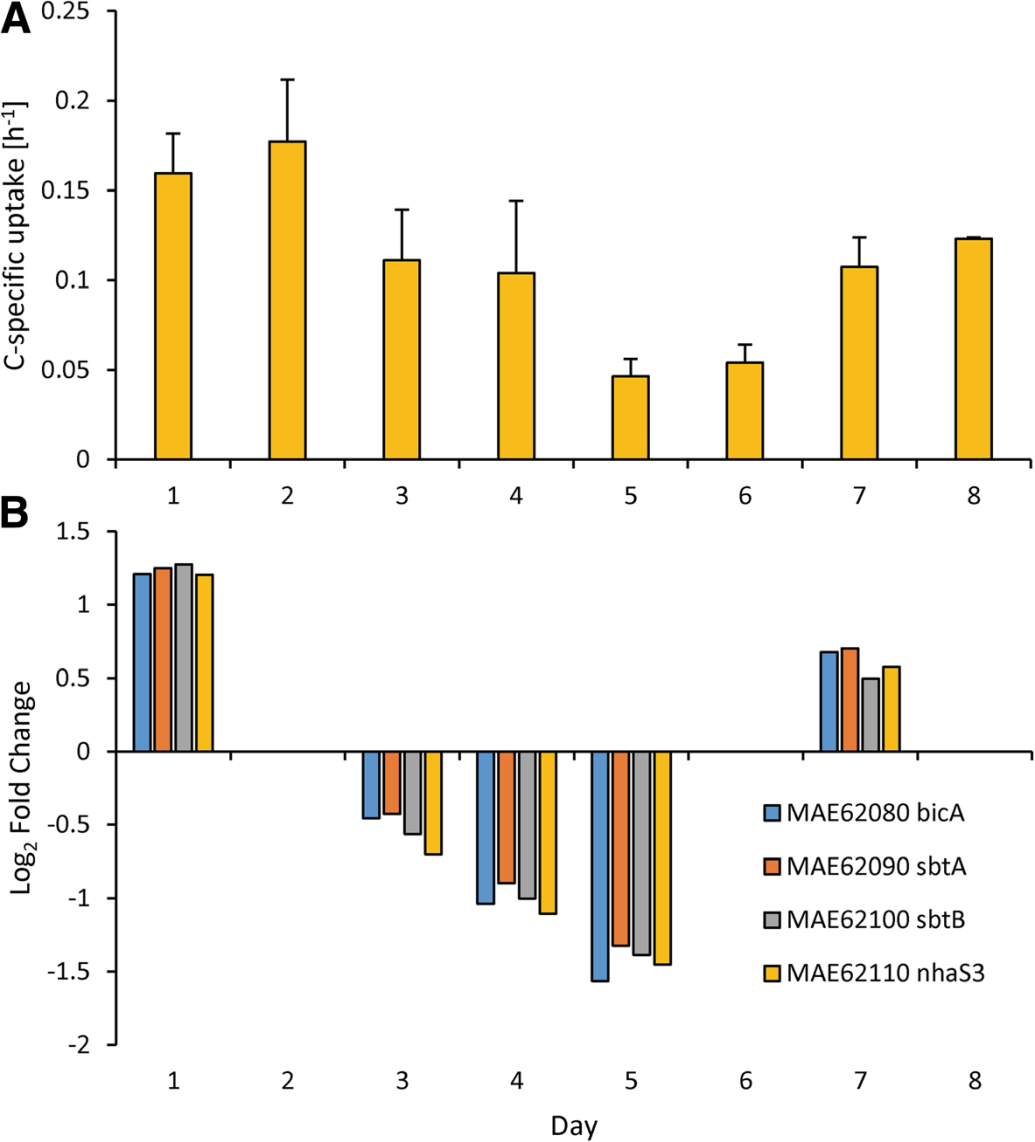
Bicarbonate specific uptake rates and bicarbonate transporter gene expression patterns. Bicarbonate specific uptake rates (**a**) and gene expression of the bicarbonate transport system (**b**) during the experiment. *Error bars* are the standard deviation between biological replicates (*n* = 3)

Uptake of nitrogenous compounds varied both between N compounds (nitrate, ammonium, and urea) and with time and generally matched expression patterns of associated N-specific transporters. For instance, uptake rates of nitrate were static on days 1–5 (averaging 3.34 ± 0.91 × 10^−3^ hr^−1^) but increased six-fold after N restoration on day 6 to 2.04 ± 1.11 × 10^−2^ hr^−1^ (Fig. 6a). Similarly, a nitrate/nitrite transporter gene set (*nrtBCD*) was upregulated by 0.95 ± 0.24 log_2_ fold change during days 2–5 but increased by three-fold to 2.62 ± 0.07 log_2_ fold change on day 6 after nitrate was added (Fig. 6b). Uptake rates of nitrate and expression of these nitrate/nitrite transporters were highly correlated (*p* <0.001; Fig. 6a, b). In contrast, another set of these nitrate/nitrite transporters (*nrtBCD*, MAE14790, 14780, 14770) was downregulated when nitrate levels decreased (Fig. 6c). Uptake rates of ammonium and urea increased as cultures became more N limited but decreased once N was restored (Figs. 7a and 8a) and ammonium and urea transporters (*amt* and *urtABCDE*, respectively) were upregulated during N limitation, but returned to control levels once N was restored (Figs. 7b and 8b). Ammonium and urea uptake constituted an increasing percentage of overall N uptake during N limitation, from 72 % of uptake to 94 % of uptake by day 5. After nitrate was restored, nitrate constituted half of the total measured N uptake (47 % on day 6; Additional file 5: Figure S4). Bicarbonate uptake decreased as cultures became more N limited and increased once N was restored (Fig. 9a). Expression patterns of genes within the bicarbonate uptake system (MAE62080, *bicA*; MAE62090, *sbtA*; MAE62100, *sbtB*; and MAE62110, *nhaS3*) [49] mirrored uptake patterns with gene expression downregulated on days 2–5 (Fig. 9b) but upregulated on day 7 (Fig. 9b) as uptake increased (Fig. 9a). As with nitrate uptake, uptake of bicarbonate and expression of the bicarbonate transporter gene set were highly correlated during this study (*p* <0.001, Fig. 9).

## Discussion

During N deprivation, cyanobacterial cells undergo numerous physiological changes including chlorosis [50], increased uptake of alternative forms of N, and an increased reliance upon internal stores of C & N [51]. There is also a reduction in total protein synthesis, although proteins required for acclimation to nutrient stress may be upregulated [51, 52]. Furthermore, there is evidence that N-starved *Microcystis* cells decrease their cellular concentrations of microcystin [11, 14, 15] and decrease expression of genes responsible for synthetase of this toxin [11]. Here we sought to build on these prior observations by documenting the daily, global transcriptional patterns of *Microcystis aeruginosa* as well as N and bicarbonate uptake rates as cells entered and exited an N deprived state.

Cultures of *Microcystis aeruginosa* LE-3 exposed to low N conditions rapidly drew down external concentrations of nitrate (from 50 μM initial concentrations to <1 μM by day 2, Table 1) and exhibited multiple physiological characteristics of N limitation, including lower photosynthetic efficiency and cellular chlorophyll *a* content (Fig. 1) [20, 50, 51]. In parallel, numerous genes involved in N acquisition and transport increased in transcript abundance (Fig. 2), while genes encoding photosynthesis and respiration, carbon acquisition, lipid metabolism, and amino acid biosynthesis decreased in transcript abundance (Figs. 4, 5 and Additional file 4: Figure S3). During N deprivation, cyanobacteria may rely on reserve materials to meet cellular demand, including photosynthetic pigments, lipids, and the N storage product cyanophycin [51, 53]. Consistent with this, as *Microcystis* cells became more N limited, the gene encoding a phycobilisome degradation protein was increasingly upregulated (*nblA*, MAE02520) and photosynthetic efficiency declined, a result observed in other cyanobacteria [54, 55]. Previous research has indicated that cyanophycin levels are generally low in rapidly growing cells but increase when nitrogen-deficient cyanobacteria are exposed to N excess or when cells are starved for light, phosphorus, or sulfur [53, 56, 57]. In this study, the expression of genes involved in the breakdown (MAE29150, *cphB*) and synthesis (MAE27490, *cphA*) of cyanophycin suggested that during N-deprivation *Microcystis* was synthesizing cyanophycin (upregulation of *cphA* on days 3 and 4) and not degrading it (downregulation of *cphB*). Cyanophycin has been shown to be synthesized during declining N conditions in *Anabaena cylindrica* and *Synechocystis* 6308 but completely consumed when all exogenous N was depleted by these cyanobacteria [58]. As suggested by these authors, cyanophycin may act as a dynamic N reservoir in cyanobacteria, including *Microcystis*, allowing cells to balance internal demand with changing external supplies of N.

Lipids can be divided into two main groups: nonpolar (acylglycerols, sterols, fatty acids) and polar lipids (phosphogylcerides, gylcosylglycerides) [59]. Polar lipids and sterols are important structural components of cell membranes while nonpolar lipids can be important for energy storage and therefore play an important role in tolerance and survival of organisms to a variety of stressors, including N deprivation [59, 60]. During N stress, cyanobacteria appear to reduce lipid and fatty acid inventories, but not composition [61–63]. Reduced lipid content under N stress is likely attributed to degradation of thylakoid membranes during the breakdown of the photosynthetic apparatus, which has been observed in numerous cyanobacteria [56, 63, 64]. Although lipid inventories were not quantified in this study, many of the genes involved in lipid and fatty acid metabolism were downregulated, suggesting lipid production was halted. However, some genes in the fatty acid, phospholipid, and sterol metabolism functional group were upregulated suggesting a more complex response N stress. For instance, a gene encoding a putative sterol C5-desaturase (MAE 29450) had increased transcript abundance during N stress. Sterol desaturases are thought to be involved in sterol biosynthesis [65], however there is still much debate as to whether cyanobacteria produce sterols (a common component of eukaryotic membranes), despite many sequenced genomes contain genes that closely match at a sequence level to sterol biosynthesis genes [66]. Further, a gene encoding diacylglycerol kinase (MAE 39670), thought to act as signaling compound with involvement of regulating lipid metabolism [67], was also upregulated. Clearly, N stress elicits a complex response at the cellular level, with potential loss and rearrangement of membrane structures.

During this study, the responses of different gene groups to the restoration of N differed. Nearly every photosynthetic gene that was downregulated on day 5 (the peak of N-deficiency as defined by photosynthetic efficiency) was expressed at normal levels 24 h after N had been restored. In contrast, most of the N metabolism genes, including the transporters of nitrogenous nutrients, were differentially expressed until day 7, nearly 48 h after N restoration began. Similar patterns were observed in the uptake of nitrate. While it is possible that an environmentally unrealistic, larger addition of N may have elicited a swifter change in the expression of N transporters, the 25 μM nitrate added was enough to return expression of most photosynthetic genes to levels similar to the control in less than 24 h and thus indicates the different physiological responses of these two gene groups in *Microcystis*. This rapid recovery of photosynthesis has been observed in other cyanobacteria at a physiological and genetic level [30, 56]. Indicators of photosynthetic activity including photosynthetic efficiency (F_v_/F_m_), chlorophyll *a* content per cell, and uptake of bicarbonate were largely unchanged until 48 h after N restoration evidencing a 24 h lag between altered gene expression and protein synthesis and/or physiological response. Krasikov et al. [30] similarly reported that N transporter genes in *Synechocystis* recovered 6–12 h later than photosynthesis genes upon N restoration. The continued need for N (continued upregulation of N transporters) likely reflects the continued cellular N demand during recovery from N limitation [56, 68]. Beyond photosynthetic and N transport and metabolism genes, other gene groups such as microcystin synthetase and lipid metabolism had individual genes that were no longer differentially expressed on days 6 and/or 7 indicating that there was not a universal transcriptional pattern in *Microcystis* as it recovered from N-limitation and emphasizing the importance of a time series-based approach to fully understand physiological responses.

Uptake rates of individual nitrogenous compounds closely mirrored expression of N-specific transporters during this study. As cells became more N starved, they preferentially and progressively increased uptake of ammonium and urea (Additional file 5: Figure S4) and upregulated the transcription of associated transporters (Figs. 7 and 8). Although uptake was not correlated with gene expression for ammonium and urea, upregulation of these transporters occurred and there was a >4-fold increase in ammonium and urea uptake rates. When nitrate was restored, ammonium and urea transporters returned to transcript levels similar to the control within 48 h and ammonium and urea uptake rates declined. In contrast, expression of some nitrate transporter genes and nitrate uptake rates were strongly correlated. During N starvation, expression of one nitrate transport gene set (*nrtBCD*, MAE18880, 18890, 18900) and uptake rates of nitrate remained static whereas restoration of nitrate was accompanied by the expression of genes for this nitrate transport gene set increasing 3-fold and nitrate uptake rates increasing 6-fold. These suggest that N starved cells grown in the absence of nitrate preferentially assimilate reduced forms of N (ammonium and urea) until nitrate becomes available. These expression patterns differ from *Synechocystis* which, under N starvation, upregulated all N transporters (nitrate, ammonium, urea and amino acids) [30]. In contrast, *Microcystis* tailored its response more closely to available N supplies. When limited by N, *Microcystis* turns on all N transporters but when one type of N is available (here nitrate), *Microcystis* increased production of the associated transporter, decreasing the production of other transporters (Figs. 6, 7 and 8). This contrasts with patterns of P-scavenging genes in *Microcystis* whose expression patterns were down- rather than upregulated when P-limited cultures were refed either inorganic or organic phosphorus [69].

During this study we believe we have identified high and low affinity nitrate transporters in *Microcystis*. During nitrogen limitation, one set of nitrate transporters (*nrtBCD*, MAE18880, 18890, 18900) was upregulated, correlating with nitrate uptake rates (Fig. 6a, b), while another set (*nrtBCD*, MAE14790, 14780, 14770) was downregulated (Fig. 6c). In our prior work [11], both sets of nitrate transporters were upregulated under nitrogen limitation (low N), but the degree of upregulation varied with one set of transporters (*nrtBCD*, MAE18880, 18890, 18900) having larger fold change values. In addition, in treatments in this study where there was higher concentrations of nitrate (>2.5 μM), the transporter set MAE14790, 14780, 14770 had much higher fold change values (between 4.56 and 23.18) than MAE18880, 18890, 18900 (between 2.59 and 4.16). These expression patterns suggest differences in transport affinity with the high affinity transport set (active under N-limitation) represented by MAE18880, 18890, 18900 and the low affinity set (active under N-replete conditions) represented by MAE14790, 14780, 14770. The *nrtABCD* nitrate uptake system has been previously described as a high-affinity transport system in *Synechococcus* [70] and high-affinity and low-affinity nitrate transport systems have been described in higher plants [71] while multiple copies of nitrate transporters have been detected in marine phytoplankton [72]. However, this is the first documentation of the existence of potential differences in the functionality of nitrate uptake genes in *Microcystis*. Further study will be needed to definitively confirm how differences in expression patterns may be related to differences in affinity among these nitrate transporters.

To our knowledge, the relationship between the expression of transporter genes and the uptake of corresponding target compounds (as measured here via isotopic tracers) has never been documented in cyanobacteria. Using a combined proteomic-transcriptomic approach, Wurch et al. [73] found that increased transcript abundance of P-transporter genes in a pelagophyte alga led to immediate increases in encoded transporter proteins. Cessation of gene expression, however, did not lead to an immediate reduction in transporter proteins suggesting that protein degradation can lag behind gene expression which is more responsive to environmental conditions [73]. During this study, after N was restored, N transporters took greater than 24 h to be downregulated and for N uptake rates to decline. Furthermore, since not all expression patterns correlate to uptake (in this study) or protein abundance [73], equating traditional cellular analyses of physiological state (such as APA, uptake, or F_v_/F_m_) to actual cellular status can be problematic. This illustrates the value of time course studies exploring gene expression and physiological responses as a means to provide detailed insight regarding the cellular physiology of microbes.

As cells become N limited, they must reduce their photosynthetic pigment inventory as metabolic capacity slows [51] in order to reduce excess energy and thereby avoid photo-damage [74]. In cyanobacteria, this is largely achieved via the degradation of phycobiliproteins as well as chlorophyll *a* [20, 51]. The gene responsible for the breakdown of phycoblisomes, *nblA* (MAE02520), was upregulated during N deprivation (Fig. 4) and genes involved in the production of the various photosystems were downregulated (Fig. 4). In addition to preventing photo-damage, the breakdown of phycobiliproteins may also provide internal N and C sources, as these pigments can constitute 50 % of soluble cellular protein in cyanobacteria [51, 75].

Beyond reduction of photosynthetic pigments, cyanobacteria must also tune their cellular C and N balance during N deprivation. Accordingly, numerous carbon concentrating mechanisms (ccm’s) were downregulated along with a bicarbonate transport system under low N (*bicA*, *sbtA*, *sbtB*, and *nhaS3*) [49] and the uptake of bicarbonate was significantly correlated with the expression of these bicarbonate transport genes that were up and down regulated under high and low N, respectively. These gene expression patterns have been observed in *Synechocystis* where many carbon concentrating mechanism genes were downregulated in response to N deprivation [30]. This reduction in uptake and expression of transport genes is likely due to a reduced need for cellular C due to lowered metabolism and photosynthesis as well as catabolism of internal stores of C from phycobiliproteins. In support of this hypothesis, the rate of inorganic carbon (C_i_) uptake has been found to be intimately linked with photosynthesis as PSI energy is thought to drive C_i_ transport [76] and bicarbonate transport rates were strongly downregulated by N starvation during this study. Therefore, the degradation of the photosynthetic apparatus in response to N deprivation leads to reduction of carbon concentrating and uptake.

In addition to the above mentioned metabolic changes occurring due to fluctuating C:N balance during N deprivation, our prior work has shown that under low N conditions, *Microcystis* contains lower levels of microcystin and downregulates some genes within the microcystin synthetase (*mcy*) cassette [11]. It was recently proposed that high C:N ratios will stimulate microcystin production and that an imbalance between 2-oxoglutarate (a compound intimately linked to maintaining C:N balance in the cell) and ammonium regulates microcystin synthesis [77]. In the present study, as cells entered N limitation (high C:N ratio), the microcystin content of cultures significantly declined (by 50 %), *mcy* genes involved in tailoring and transport were upregulated (*mcyHIJ*), genes involved in peptide synthesis were downregulated (*mcyACDE*), and some *mcy* genes displayed no change relative to the control condition (*mcyBFG*; Fig. 5). These findings are generally consistent with prior studies where under N limitation, the *mcyABCDEF* genes were downregulated, with all but *mcyF* being involved in peptide synthesis [11] suggesting that microcystin production relates more to N supply than C:N balance. Further, these changes in mcy transcript abundance suggest a partitioned response to N deprivation whereby genes involved in peptide synthesis are downregulated but genes involved in the tailoring and export of microcystin are upregulated. In addition, upon N restoration, transcripts of the *mcyCDE* genes (involved in peptide synthesis) were restored to control levels and concentrations of microcystin increased by 2-fold. The observed expression patterns demonstrate that all microcystin synthetase genes do not respond similarly to physiological changes within the cell and that the genes involved in peptide synthesis play a central role in modulating cellular microcystin content and are responsive to N availability. When exploring microcystin gene expression responses to environmental or physiological changes, most studies to date have focused on a single peptide synthesis gene *(mcyA, B, D, or E)* [78–84], an approach that does not provide a global view of microcystin synthesis. For example, had this study focused solely on *mcyB* (as in Ginn et al. [82] and Beversdorf et al. [83]), we would have concluded there was no interaction with N, as this gene was not differentially expressed during the experiment. Furthermore, time is a significant factor to consider as expression of each gene varied over time. Therefore, conclusions based on the expression of one peptide synthetase gene or a single time point may be biased by the response of the individual gene to environmental conditions or physiological cues.

Currently there are over 89 identified congeners of microcystin [85] and some strains of *Microcystis* are thought to incorporate different amino acids into the microcystin structure leading to changes in congener and relative toxicity [86]. *Microcystis* may also incorporate different amino acids during light and dark periods based upon amino acid availability that may alter the variant of microcystin synthesized [87]. Furthermore, microcystin is a N-rich compound, containing seven N atoms in its core ring with additional N in the variable L-amino acid residues [88]. It is therefore an N-expensive compound to produce. Production of this compound has been shown to decrease as cellular N-stores and the levels of exogenous N are reduced (N-deprivation and starvation) [5, 14, 15] and increase when N becomes available [12, 13, 15]. The upregulation of the two genes encoding tailoring enzymes (*mycIJ*) during N deprivation may reflect active modification of microcystin to different congeners. If this were true, it could have implications on overall toxicity [89, 90]. However, the upregulation of these tailoring genes could also represent active disassembly of microcystin, perhaps in an effort to meet N demand during N deprivation. In this hypothesis, microcystin may act as an N reserve material to allow toxic strains to persist during times of N stress. Future work characterizing congener specific changes during N deprivation and/or investigations into the fate of microcystin may clarify this.

An understanding of the function of microcystin remains elusive, despite decades of research on this compound. There have been numerous theories relating the role of microcystin to quorum sensing, inhibition of competitors, managing oxidative stress, or grazing deterrence [87, 91–93] and it has been suggested that the compound is not essential for growth [94]. Three of these theories (quorum sensing, inhibition of competitors, or grazer deterrence) are each predicated upon the compound being actively exported from the cell and the *mcyH* gene is thought to be involved in transport of microcystin outside the cell as it bears similarity to an ABC transporter [95]. Protein location prediction using PSORTb [96], however, predicts *mcyH* is located within the inner membrane of the cell suggesting that *mcyH* exports microcystin to the periplasmic space and not out of the cell. In support of this, Rohrlack and Hyenstrand [87] used a ^14^C tracer to track the location and fate (either exported from the cell or metabolized) of microcystin under various light conditions and found no evidence of export or intracellular breakdown under these conditions. They did not, however, assess cultures under nutrient limitation, a condition which commonly occurs during blooms [6, 33, 97]. In the current study, the microcystin transporter *mcyH* was upregulated under N limitation potentially supporting the hypothesis that microcystin is a quorum sensing compound as there were no grazers or competing algae present to induce an export response. However, there may also be internal cues triggering the export of microcystin, such as internal N-status or stress. If indeed cells are exporting microcystin during N limitation, this would be a counterintuitive response since, as mentioned earlier, microcystin is an N-rich compound and cells would, in effect, be furthering their N limitation at a time of greatest need for the nutrient. Regardless, peptide synthesis genes were downregulated and concentrations of microcystin declined during N deprivation while transport and modification genes were upregulated, suggesting this compound may have an important extracellular function.

## Conclusions

In summary, during N deprivation, *Microcystis* downregulated genes involved in photosynthesis, respiration, carbon acquisition, lipid metabolism, and amino acid biosynthesis while expression of genes relating to N acquisition and transport was upregulated. As N stress intensified, the response increased in both the strength of expression and number of genes. When N was restored, many of these responses reversed. Uptake of bicarbonate, nitrate, ammonium and urea generally paralleled expression of transporters for these compounds. *Microcystis* increased uptake and transcript abundance of transporters for ammonium and urea as cells became more N limited. When nitrate was restored, nitrate transporters were strongly upregulated and nitrate was assimilated at a high rate. Further, we have shown evidence that *Microcystis* has both a high and low affinity nitrate/nitrite uptake system as one gene set was upregulated and correlated with nitrate uptake while the other set was downregulated. Lastly, while concentrations of microcystin decreased during N deprivation and increased upon N restoration, the response of genes involved in the synthesis of this compound was more complex. Genes involved in peptide synthesis (*mcyACDE*) were downregulated during N deprivation whereas genes involved genes in tailoring and transport of microcystin (*mcyHIJ*) were upregulated, suggesting this compound could serve an external function under N stress. Collectively, this study highlights the complex choreography of gene expression, cell physiology, and toxin synthesis that dynamic N levels can elicit in this ecologically important cyanobacterium.

## Additional files

Additional file 1: Table S1.
**Transcriptomic sequencing results**. The number of sequenced reads that aligned to the *Microcystis aeruginosa* NIES-843 genome using Bowtie 2 within RSEM. Each treatment has three biological replicates. (DOC 93 kb) Additional file 2: Figure S1. The number of differentially expressed genes in the low N experiment as compared to the control. Nitrogen refeed occurred on day 5. Bars in the positive axis are those genes with increased transcript abundance and those in the negative access are genes with decreased transcript abundance. Solid bars are genes exhibiting log_2_ fold change values >2. (TIFF 226 kb) Additional file 3: Figure S2. The number of genes differentially expressed (DE) for each time point of the experiment in each functional category (as defined by CyanoBase). Values in parenthesis next to the category name are the number of genes present in the category. (TIFF 303 kb) Additional file 4: Figure S3. Heat map of gene expression of genes involved in fatty acid, phospholipid and sterol metabolism. Blue colors correspond to a decrease in transcript abundance while red colors correspond to an increase in transcript abundance. White colors denote no difference from the control condition. (TIFF 1761 kb) Additional file 5: Figure S4. Nitrogen uptake of ^15^N-labeled compounds as a percentage of total N uptake. (TIFF 115 kb)
