# Supplementary material for: Daily transcriptome changes reveal the role of nitrogen in controlling microcystin synthesis and nutrient transport in the toxic cyanobacterium, Microcystis aeruginosa
Source: BMC Genomics. 2015 Dec 16;16:1068. doi: 10.1186/s12864-015-2275-9 (PMC4681089; doi:10.1186/s12864-015-2275-9)
Supplement: Additional file 1: Table S1. — Transcriptomic sequencing results. The number of sequenced reads that aligned to the Microcystis aeruginosa NIES-843 genome using Bowtie 2 within RSEM. Each treatment has three biological replicates. (DOC 93 kb) [file 12864_2015_2275_MOESM1_ESM.doc]

| **Table S1** **Transcriptomic sequencing results**. The number of sequenced reads that aligned to the *Microcystis aeruginosa* NIES-843 genome using Bowtie 2 within RSEM. Each treatment has three biological replicates. | | | | | |
| --- | --- | --- | --- | --- | --- |
| **Treatment** | **#Reads** | **Aligned 0 times** | **Aligned exactly 1 time** | **Aligned >1 time** | **Overall alignment rate** |
| NO3 Day 1 | 40,614,296 | 16,295,287 | 19,225,431 | 5,093,578 | 59.88% |
|  | 40,297,600 | 16,549,587 | 19,289,531 | 4,458,482 | 58.93% |
|  | 40,074,135 | 10,742,959 | 11,920,220 | 17,410,956 | 73.19% |
| NO3 Day 2 | 39,511,827 | 4,005,818 | 3,224,838 | 31,301,171 | 89.86% |
|  | 34,912,882 | 6,343,843 | 5,205,978 | 23,342,861 | 81.83% |
|  | 36,736,741 | 4,131,827 | 2,931,753 | 29,672,961 | 88.75% |
| NO3 Day 3 | 40,813,656 | 7,725,834 | 6,177,335 | 26,910,487 | 81.07% |
|  | 36,638,931 | 16,661,024 | 15,722,853 | 4,247,054 | 54.53% |
|  | 35,424,926 | 16,457,812 | 14,321,017 | 4,646,897 | 53.54% |
| NO3 Day 4 | 42,833,408 | 19,819,814 | 18,193,144 | 4,820,450 | 53.73% |
|  | 38,909,097 | 18,039,994 | 16,436,728 | 4,432,375 | 53.64% |
|  | 37,476,921 | 17,681,123 | 15,268,305 | 4,527,493 | 52.82% |
| NO3 Day 5 | 30,602,342 | 14,754,630 | 12,668,872 | 3,178,848 | 51.79% |
|  | 35,386,038 | 17,168,992 | 15,075,931 | 3,141,115 | 51.48% |
|  | 30,684,847 | 14,933,358 | 12,940,781 | 2,810,708 | 51.33% |
| NO3 Day 6 | 42,410,095 | 17,273,734 | 20,771,737 | 4,364,624 | 59.27% |
|  | 39,910,356 | 17,148,918 | 18,480,358 | 4,281,080 | 57.03% |
|  | 38,214,156 | 17,041,871 | 17,210,671 | 3,961,614 | 55.40% |
| NO3 Day 7 | 40,390,091 | 16,526,147 | 20,388,908 | 3,475,036 | 59.08% |
|  | 37,049,025 | 14,899,950 | 19,064,661 | 3,084,414 | 59.78% |
|  | 36,581,729 | 15,536,946 | 16,727,601 | 4,317,182 | 57.53% |
| NO3 Day 8 | 36,392,263 | 14,918,420 | 17,855,386 | 3,618,537 | 59.01% |
|  | 33,487,546 | 15,356,861 | 13,151,785 | 4,978,900 | 54.14% |
|  | 36,063,108 | 16,472,270 | 14,787,586 | 4,803,332 | 54.32% |
| Control Day 4 | 36,553,580 | 14,977,018 | 16,767,328 | 4,809,234 | 59.03% |
|  | 42,689,139 | 18,009,540 | 19,589,812 | 5,089,787 | 57.81% |
|  | 42,726,343 | 17,985,778 | 19,221,469 | 5,519,096 | 57.90% |
