# Supplementary figures and images for: Daily transcriptome changes reveal the role of nitrogen in controlling microcystin synthesis and nutrient transport in the toxic cyanobacterium, Microcystis aeruginosa
Source: BMC Genomics. 2015 Dec 16;16:1068. doi: 10.1186/s12864-015-2275-9 (PMC4681089; doi:10.1186/s12864-015-2275-9)

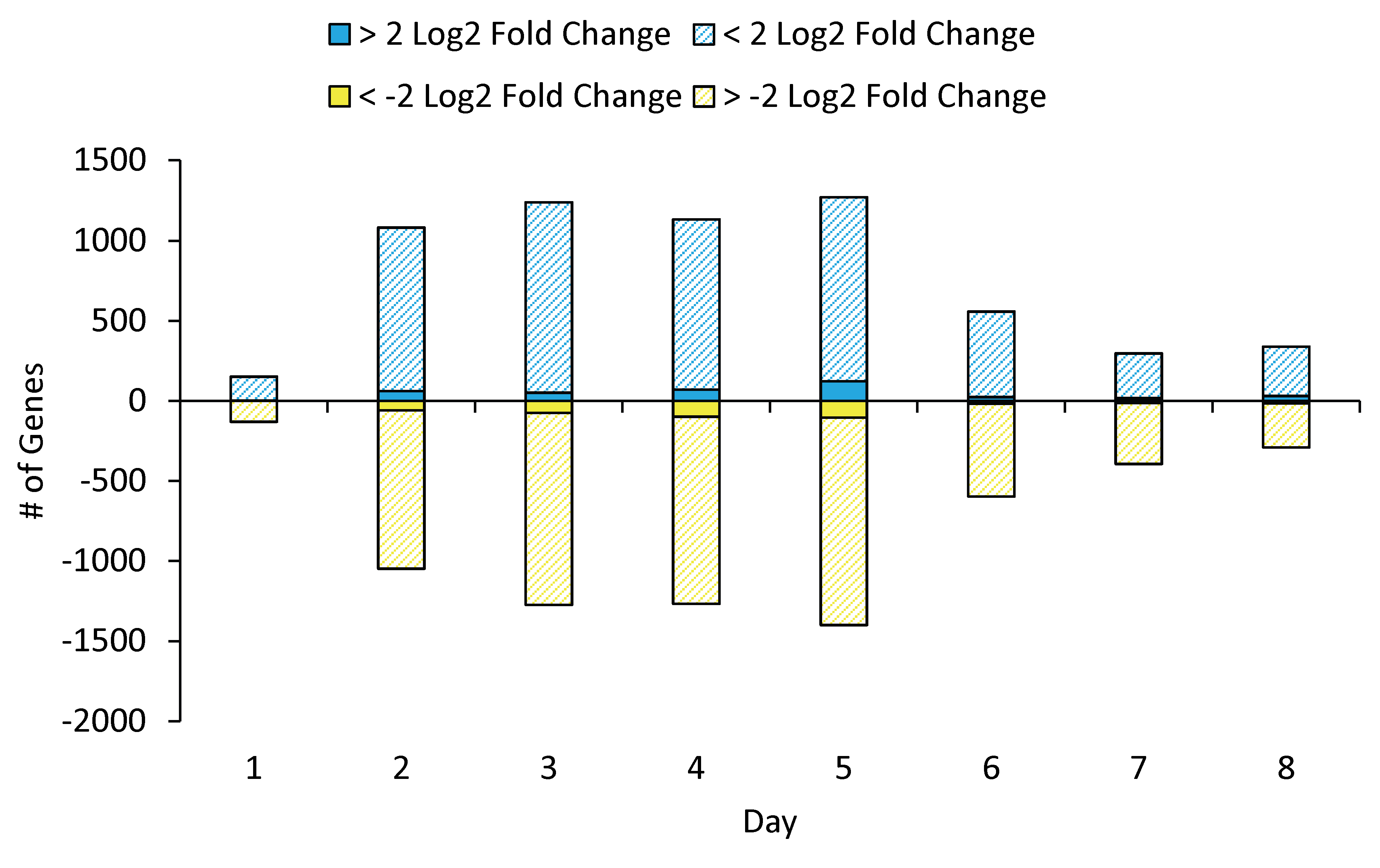

Supplement: Additional file 2: Figure S1. — The number of differentially expressed genes in the low N experiment as compared to the control. Nitrogen refeed occurred on day 5. Bars in the positive axis are those genes with increased transcript abundance and those in the negative access are genes with decreased transcript abundance. Solid bars are genes exhibiting log2 fold change values >2. (TIFF 226 kb) [file 12864_2015_2275_MOESM2_ESM.tiff]

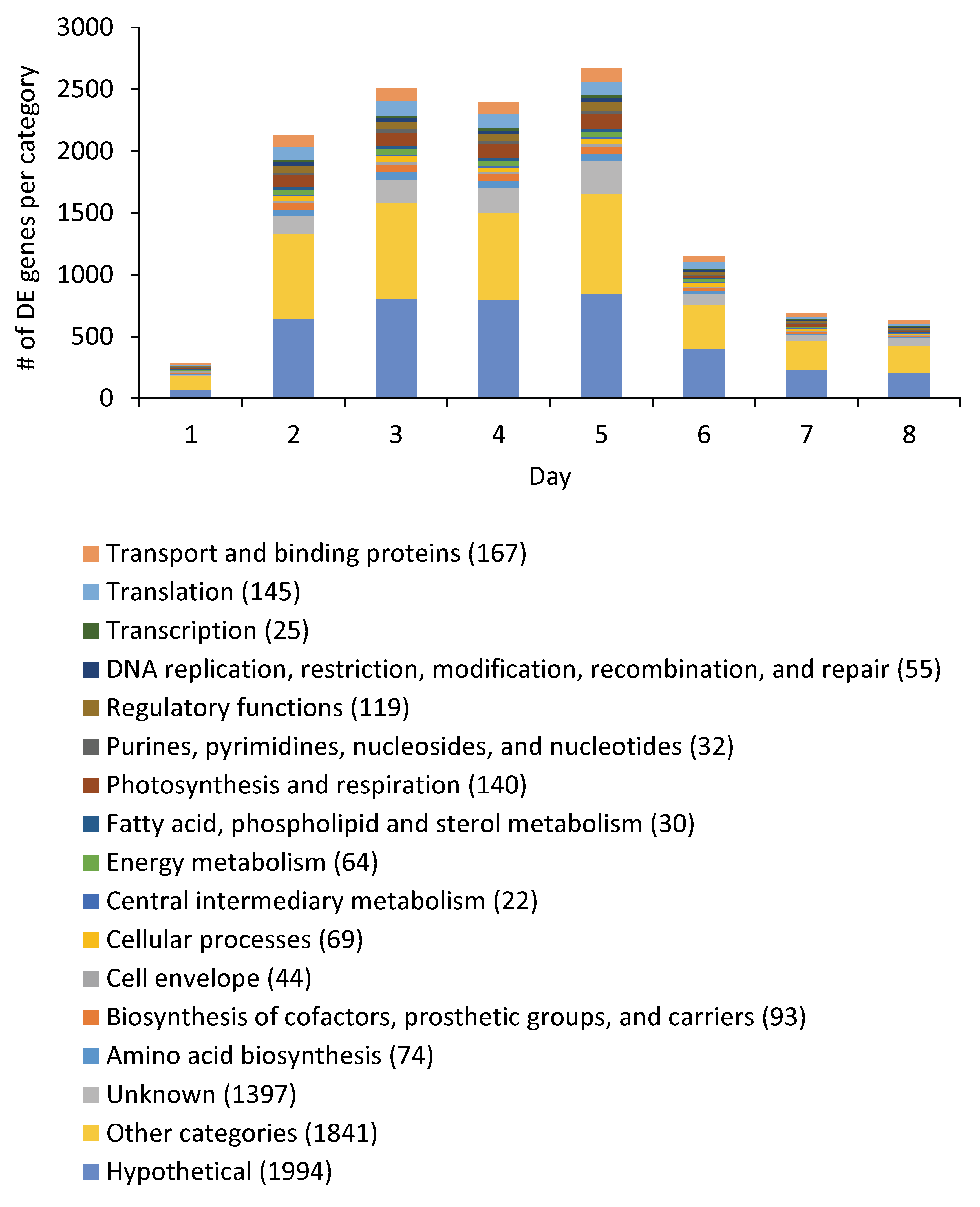

Supplement: Additional file 3: Figure S2. — The number of genes differentially expressed (DE) for each time point of the experiment in each functional category (as defined by CyanoBase). Values in parenthesis next to the category name are the number of genes present in the category. (TIFF 303 kb) [file 12864_2015_2275_MOESM3_ESM.tiff]

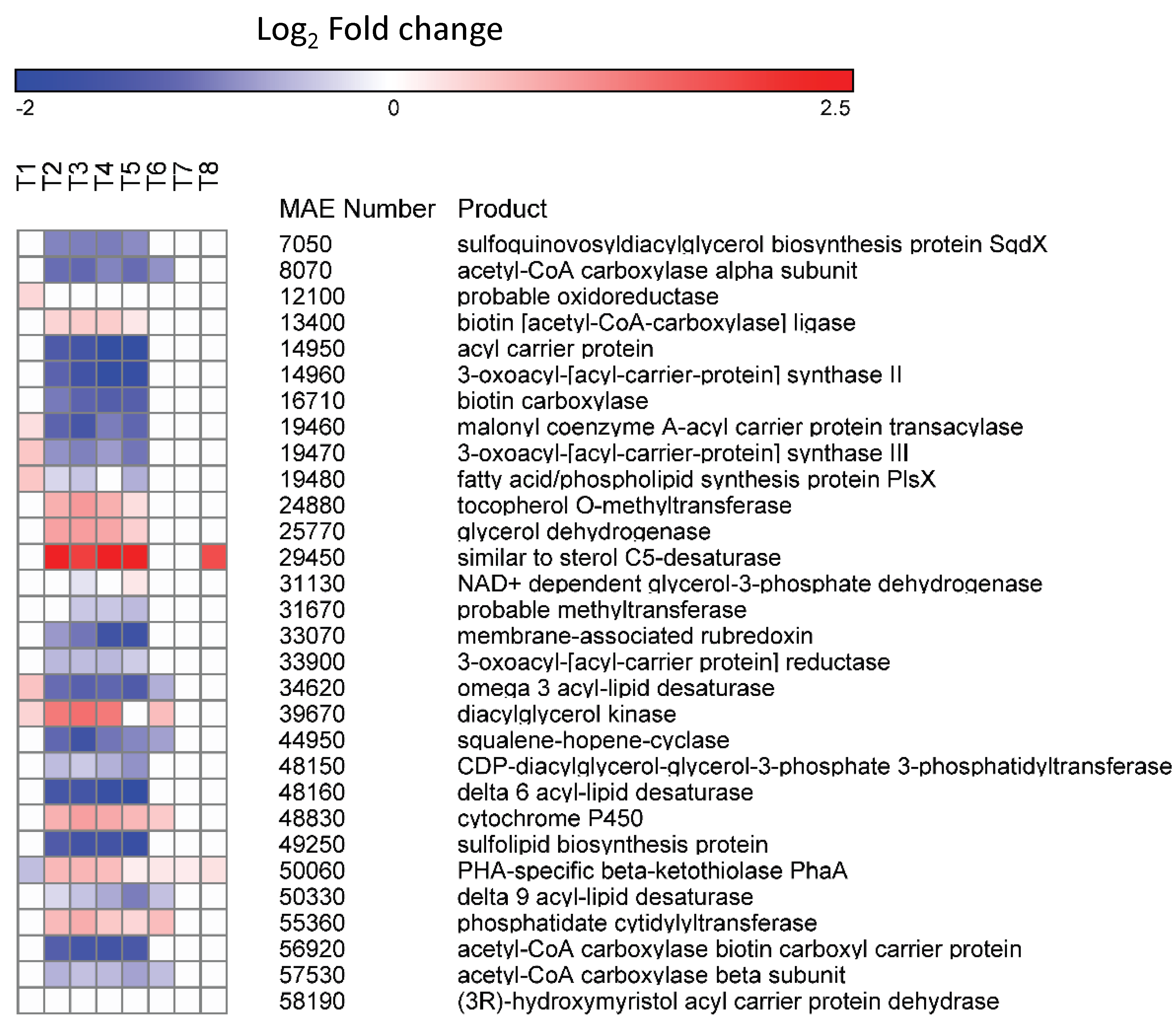

Supplement: Additional file 4: Figure S3. — Heat map of gene expression of genes involved in fatty acid, phospholipid and sterol metabolism. Blue colors correspond to a decrease in transcript abundance while red colors correspond to an increase in transcript abundance. White colors denote no difference from the control condition. (TIFF 1761 kb) [file 12864_2015_2275_MOESM4_ESM.tiff]

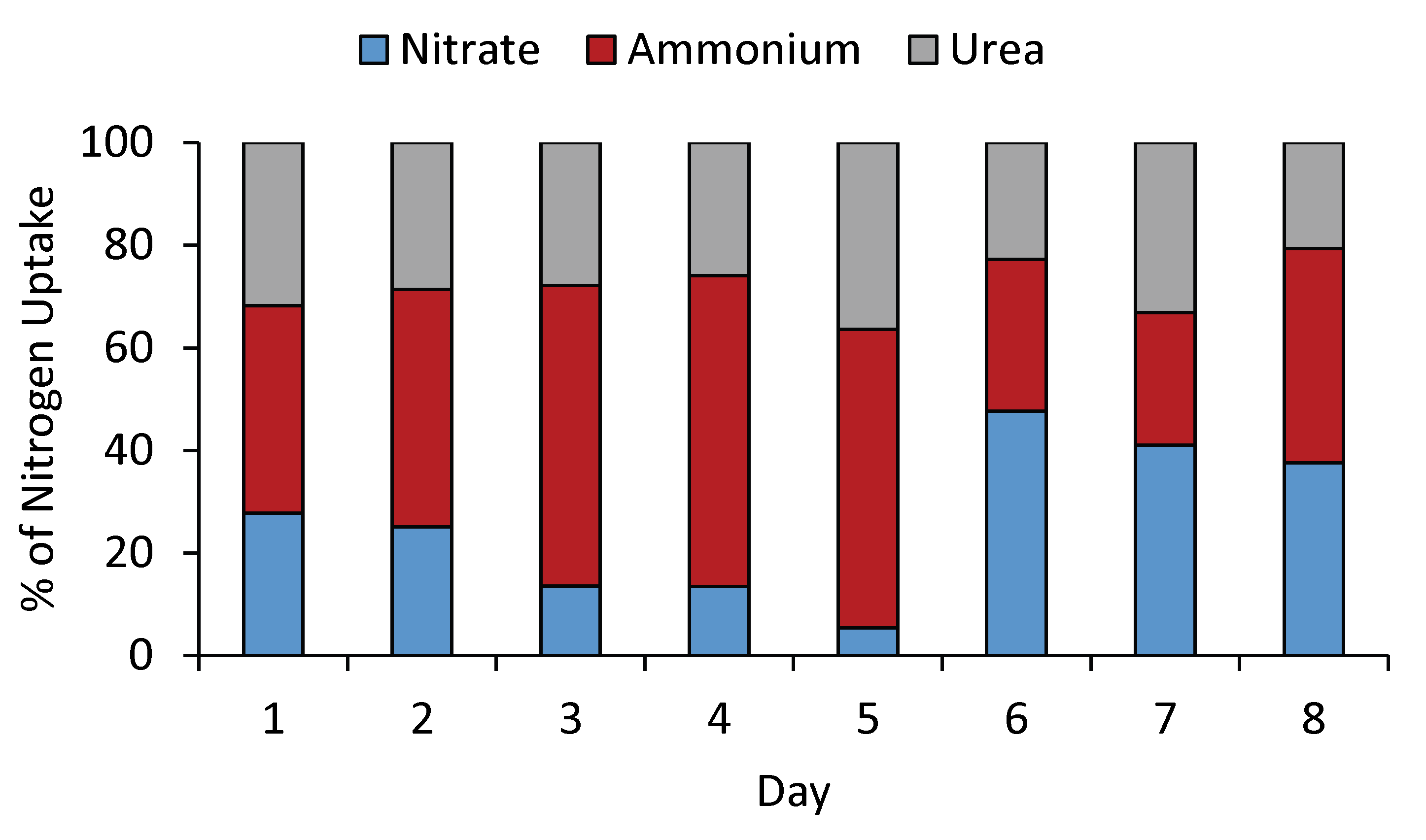

Supplement: Additional file 5: Figure S4. — Nitrogen uptake of 15N-labeled compounds as a percentage of total N uptake. (TIFF 115 kb) [file 12864_2015_2275_MOESM5_ESM.tiff]
